# Lymphocyte may be a reference index of the outcome of cancer patients with COVID-19

**DOI:** 10.18632/aging.202741

**Published:** 2021-03-18

**Authors:** Wei Zhang, Yuan Gao, Guoqing Hu, Qian Chu, Xun Yuan

**Affiliations:** 1 Department of Oncology, Tongji Hospital of Tongji Medical College, Huazhong University of Science and Technology, Wuhan, People's Republic of China

**Keywords:** COVID-19, cancer, survival time, cytokines, lymphocytes

## Abstract

Background: The novel coronavirus infectious disease (COVID-19) is an international concern as it spreads through human populations and across national and international borders.

Methods: In this retrospective study, we consecutively included all cancer patients who had been identified as having a nucleic acid-confirmed COVID-19 from two designated hospitals in Wuhan, China. COVID-19 patients without cancer were also enrolled for comparison. The clinical data were gathered from the medical records from Jan 14 to March 12, 2020.

Results: Among the 117 cancer patients diagnosed with COVID-19, the median age was 63 years and 48.7% were male. Male sex, hematologic cancer, dyspnea on admission, and anti-cancer therapies were associated with an increased risk of mortality in cancer patients with COVID-19. We found that elevated levels of TNF-α, IL-2R, IL-6, and IL-8 were associated with a poorer prognosis in cancer patients with COVID-19, but no statistically significant association was found in patients without cancer. In addition, IL-2R and IL-6 markedly decreased in cancer patients who recovered from COVID-19. However, lymphocyte subsets were diminished in cancer patients who died from COVID-19, including total T cells, total B cells, helper T (Th) cells and suppressor T (Ts) cells.

Conclusions: Cancer patients with COVID-19 were associated with high mortality (23.9%). A decrease in lymphocyte subsets and higher levels of cytokines were associated with a higher risk of severe outcome and could be utilized as the reference index to predict the survival outcome of cancer patients with COVID-19.

## INTRODUCTION

In December 2019, several pneumonia cases of unknown aetiology were identified in Wuhan, China [1]. A new enveloped RNA betacoronavirus known as the severe acute respiratory syndrome coronavirus 2 (SARS-CoV-2) has been recognized as the pathogen of 2019 novel coronavirus disease (COVID-19). As of June 28, 2020, more than 10 million laboratory-confirmed cases had been recorded globally.

Coronaviruses mainly cause respiratory tract infections, such as Middle East respiratory syndrome coronavirus (MERS-CoV) and severe acute respiratory syndrome coronavirus (SARS-CoV) [2], which have resulted in more than 10000 cases in the past twenty years, with death rates of 37% for MERS-CoV and 10% for SARS-CoV [3, 4]. In contrast to MERS-CoV and SARS-CoV, COVID-19 has caused more deaths by multiple organ failure rather than respiratory failure, due to the widespread distribution of angiotensin converting enzyme 2 (ACE2)—the binding receptor for SARS-CoV-2—in various organs [5]. The main conditions predisposing to infection were malignancy and immune-suppressive therapies, such as chemotherapy or surgery [6]. Thus, oncological patients might be more susceptible to COVID-19 and have a poor prognosis [7].

Given the rapid spread of COVID-19, we aimed to describe the epidemiological, clinical, and laboratory parameters of cancer patients with COVID-19. Furthermore, we assessed the prognostic value of cytokines and lymphocyte subsets on the outcome of these patients.

## RESULTS

### Demographic and clinical characteristics

In the 117 cancer patients with laboratory-confirmed COVID-19 who had been hospitalized at the two designated hospitals as of March 12, 2020. 56 had received anti-cancer treatments, including surgery (16.2%), chemotherapy (19.7%), and radiotherapy (4.3%) as the most recent treatment in the last year and the others were cancer survivors in routine follow-up. The demographic and clinical parameters of the 117 cancer patients are listed in Table 1. The median age was 63 years (interquartile range, 56 to 70). A total of 48.7% were male. Lung cancer was the most common type (25.6%); less common were digestive system cancer (23.9%), breast cancer (15.4%), thyroid cancer (8.5%), urinary system cancer (7.7%), gynecological tumor (6.8%), hematologic malignancies (6.8%), sarcoma (2.6%), head and neck cancer (0.9%), and cancer of unknown primary site (1.7%). Among the overall population, 48.7% had more than one coexisting illness, including diabetes (15.4%), hypertension (30.8%), cardiovascular disease (9.4%), and chronic obstructive pulmonary disease (COPD) (14.5%). Fever was present in 71.8% of these patients on admission. Less common symptoms were cough (65.0%), myalgia (50.4%), and sputum production (39.3%); diarrhea (14.5%) was uncommon.

**Table 1:** Clinicopathological characteristics of cancer patients with COVID-19.

**Characteristics**	**All patients** **(n=117)**	**Alive** **(n=89)**		**Dead** **(n=28)**	**P value**
**Age (years)**					0.499
< 60	41 (35.0)	33 (37.1)		8 (28.6)	
≥ 60	76 (65.0)	56 (62.9)		20 (71.4)	
**Gender**					0.029
Female	60 (51.3)	51 (57.3)		9 (32.1)	
Male	57 (48.7)	38 (42.7)		19 (67.9)	
**Diagnosis (n [%])**					
Lung cancer	30 (25.6)	25 (28.1)		5 (17.9)	0.280
Breast cancer	18 (15.4)	16 (18.0)		2 (7.1)	0.166
Thyroid cancer	10 (8.5)	10 (11.2)		0 (0)	0.064
Digestive System Cancer^*^	28 (23.9)	20 (22.5)		8 (28.6)	0.509
Gynecological oncology^#^	8 (6.8)	8 (9.0)		0 (0)	0.100
Urinary system tumor^&^	9 (7.7)	4 (4.5)		5 (17.9)	0.021
Hematologic malignancies	8 (6.8)	2 (2.2)		6 (21.4)	<0.001
Sarcoma	3 (2.6)	1 (1.1)		2 (7.1)	0.079
Head and neck cancer	1 (0.9)	1 (1.1)		0 (0)	0.573
Unknown primary site	2 (1.7)	2 (2.2)		0 (0)	0.424
**Comorbidities**					0.955
Diabetes	18 (15.4)	13 (14.6)		5 (17.9)	
Hypertension	36 (30.8)	26 (29.2)		10 (35.7)	
Coronary heart disease	11 (9.4)	7 (7.9)		4 (14.3)	
COPD	17 (14.5)	12 (13.5)		5 (17.9)	
**Symptom**					
Dyspnea	54 (46.2)	32 (36.0)		22 (78.6)	<0.001
Cough	76 (65.0)	59 (66.3)		17 (60.7)	0.590
Expectoration	46 (39.3)	33 (37.1)		13 (46.4)	0.377
Malaise	59 (50.4)	45 (50.6)		14 (50.0)	0.959
Headache	10 (8.5)	6 (6.7)		4 (14.3)	0.213
Muscle ache	10 (8.5)	9 (10.1)		1 (3.6)	0.280
Pharyngodynia	7 (6.0)	7 (7.9)		1 (3.6)	0.432
Diarrhea	17 (14.5)	13 (14.6)		4 (14.3)	0.967
Fever	84 (71.8)	65 (73)		19 (67.9)	0.596

Annotation: ^*^indicating 11 intestine cancer, 4 esophageal cancer, 6 liver tumor, 7 stomach cancer; ^#^indicating 2 ovary cancer, 4 cervical cancer, 2 endometrial cancer; ^&^indicating 5 bladder cancer patient, 2 prostate cancer, 2 renal cancer.

28 (23.9%) cancer patients with COVID-19 worsened and died of multiple organ failure, while 19 (16.2%) patients without a history of cancer died due to COVID-19 (Supplementary Table 1). Male and the presence of dyspnea were more prevalent among all the patients who died of COVID-19 than those alive (cancer patients p=0.029, p<0.001; non-cancer patients p=0.006, p<0.001; respectively). Moreover, oncological patients with hematologic malignancy had the highest mortality than those with non-hematologic cancer (p<0.001). However, age and comorbidities between the two groups were similar.

### Laboratory findings

The laboratory findings on admission are shown in Table 2. Lymphocytopenia was present in 59.8% of cancer patients, thrombocytopenia in 17.9%, and leukocytosis in 13.7%. Most cancer patients had increased level of C-reactive protein (CRP); less frequent were increased levels of aspartate aminotransferase (AST), alanine aminotransferase (ALT), D-dimer. The alteration of laboratory blood tests (leukocytes, neutrophils, lymphocytes, thrombocytes, prothrombin time, activated partial thromboplastin time, CRP, procalcitonin, bilirubin and troponin) were associated with outcome of cancer patients with COVID-19 (p=0.023, p=0.002, p=0.027, p=0.045, p<0.001, p<0.001, p<0.001, p=0.003, p=0.005, p<0.001; respectively). Besides, cancer patients had more prominent laboratory abnormalities than non-cancer patients (Supplementary Table 2).

**Table 2:** Laboratory findings of cancer patients with COVID-19 on admission to hospital.

**Characteristics**	**All patients** **(n=117)**	**Alive** **(n=89)**	**Dead** **(n=28)**	**P value**
WBC, ×10^9^/L				
< 4	24/117 (20.5)	19/89 (21.3)	5/28 (17.9)	0.794
> 10	16/117 (13.7)	8/89 (9.0)	8/28 (28.6)	0.023
Neutrophil count, ×10^9^/L	3.9 (2.6, 5.6)	3.6 (2.5, 5.1)	6.3 (2.9, 8.9)	0.002
Lymphocytopenia	70/117 (59.8)	48/89 (53.9)	22/28 (78.6)	0.027
Thrombocytopenia	21/117 (17.9)	12/89 (13.5)	9/28 (32.1)	0.045
Prothrombin time, s	13 (12, 14)	13 (12, 14)	15 (13, 16)	<0.001
APTT, s	36 (27, 42)	33 (27, 41)	41 (35, 49)	<0.001
D-dimer, mg/L	1.1 (0.5, 2.9)	1.0 (0.5, 2.0)	2.5 (1.1, 7.3)	0.074
CRP	36 (7, 92)	26 (5, 56)	96 (54, 145)	<0.001
Procalcitonin	0.09 (0.05, 0.26)	0.06 (0.04, 0.15)	0.39 (0.13, 1.07)	0.003
Total bilirubin	10.1 (6.7, 13.3)	9.9 (6.4, 13.0)	10.3 (8.3, 15.7)	0.005
ALT > 40U/liter	22/117 (18.8)	19/89 (21.3)	3/28 (10.7)	0.274
AST > 40U/liter	28/117 (23.9)	19/89 (21.3)	9/28 (32.1)	0.310
Troponin, pg/mL	1.9 (0, 4.4)	1.9 (0, 2.5)	15 (1.9, 61.8)	<0.001

Annotation: Lymphocytopenia was defined as a lymphocyte count of less than 1000 per cubic millimeter. Thrombocytopenia was defined as a platelet count of less than 100,000 per cubic millime- ter. APTT indicates activated partial thromboplastin time. AST indicates aspartate aminotransferase.

**Figure 1:**
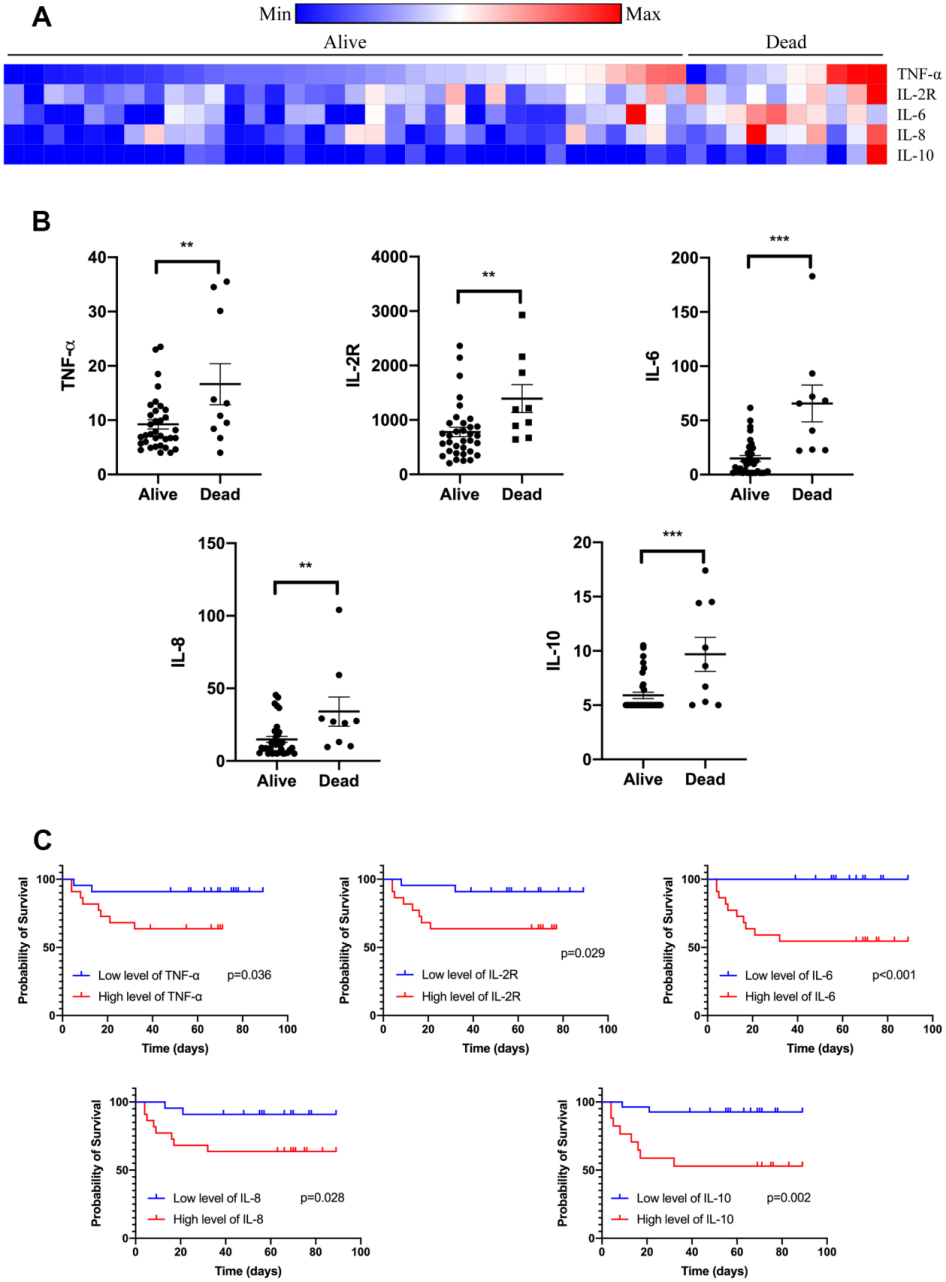
**Serum cytokines on admission of cancer patients with COVID-19.** Levels of TNF-α, IL-2R, IL-6, IL-8, and IL-10 were tested on admission of cancer patients with COVID-19 divided by the outcome (**A**). Levels of TNF-α, IL-2R, IL-6, IL-8, and IL-10 were increased in those patients who died from COVID-19 (**B**). Levels of TNF-α, IL-2R, IL-6, IL-8, and IL-10 were correlated with survival time of cancer patients with COVID-19 (**C**).

**Figure 2:**
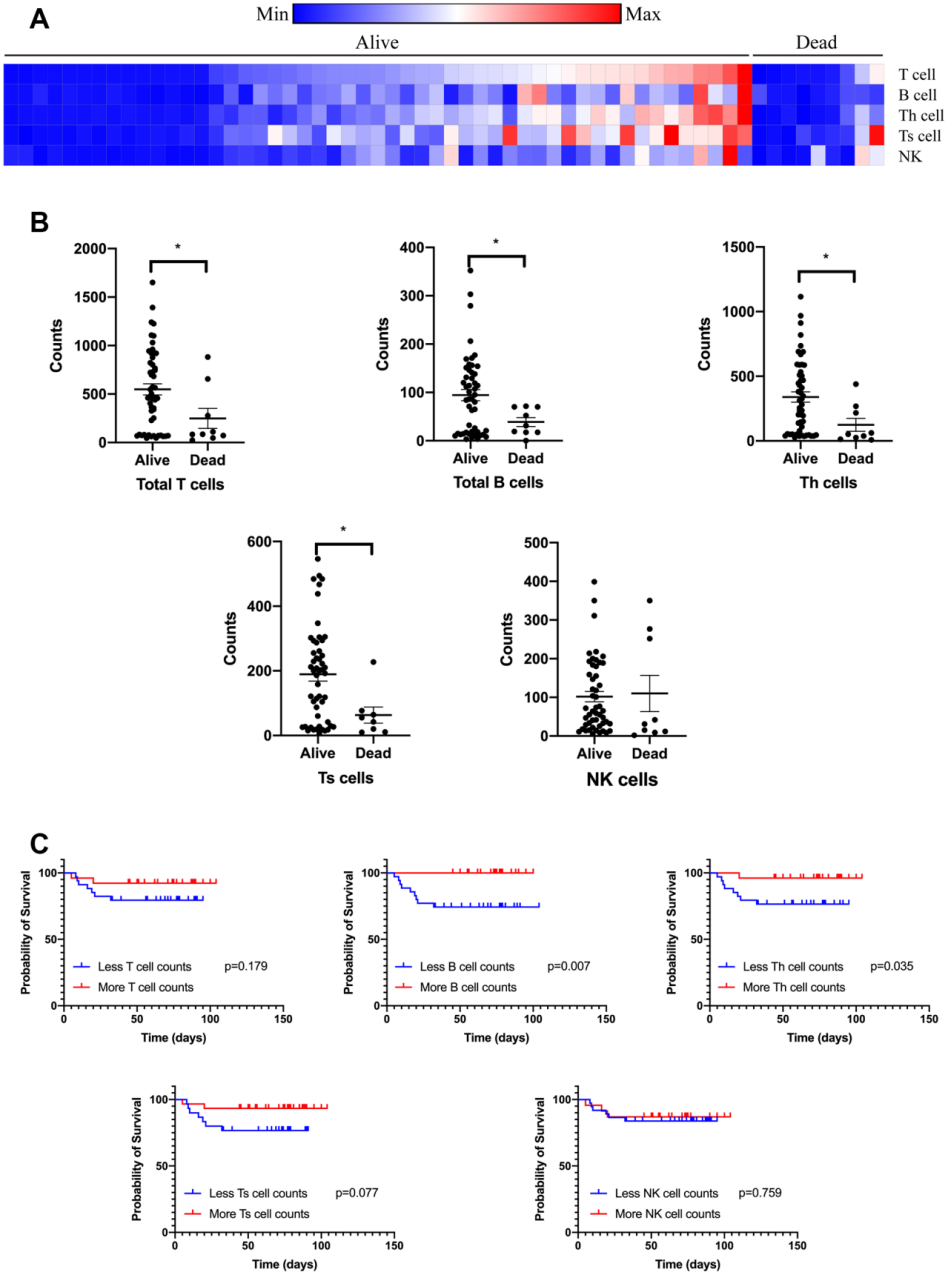
**Circulating immune cell subpopulation on admission of cancer patients with COVID-19.** Counts of T lymphocytes, B lymphocytes, Th cells, Ts cells, and NK cells were tested on admission of cancer patients with COVID-19 divided by the outcome (**A**). Counts of T lymphocytes, B lymphocytes, Th cells, and Ts cells were diminished on admission of cancer patients with COVID-19 (**B**). Count of B lymphocytes and Th cells were correlated with survival time of cancer patients with COVID-19 (**C**).

**Figure 3:**
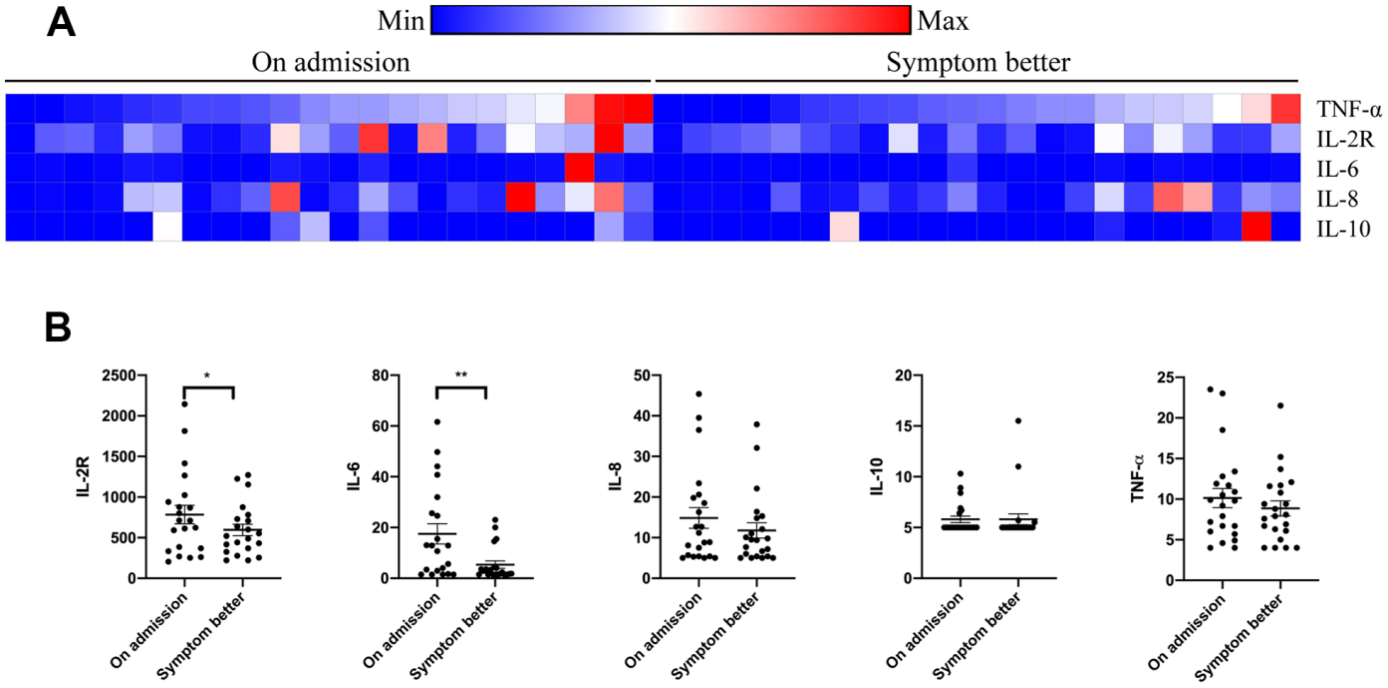
**Serum cytokines alteration in cancer patients with COVID-19 during therapy.** Levels of TNF-α, IL-2R, IL-6, IL-8, and IL-10 were tested on admission and when symptom turning better (**A**). Levels of TNF-α, IL-2R, IL-6, IL-8, and IL-10 were changed in cancer patients who recovered from COVID-19 (**B**).

Compared to those alive, all the patients who died from COVID-19 had increased levels of TNF-α, IL-2R, IL-6, IL-8, and IL-10 tested on admission (Figure 1 and Supplementary Figure 1). However, high levels of TNF- α, IL-2R, IL-6, and IL-8 did not raise the risk of death in non-cancer patients. Importantly, the levels of TNF- α, IL-2R, IL-6, IL-8, and IL-10 were significantly correlated with survival time of cancer patients with COVID-19. These odds were further proved by logistic regression model after adjusting for other risk factors, including age, gender, cancer type, comorbidities, symptoms, and anti-cancer treatments (Table 3). In addition, T lymphocytes, B lymphocytes, T helper (Th) cells, and T suppressor (Ts) cells were diminished in cancer patients with a primary composite end-point event of death (Figure 2). Cancer patients with more B cells and Th cells had longer survival time than those with less B cells and Th cells (p=0.007, p=0.035; respectively). We further examined if the levels of cytokines were changed when cancer patients recovered from COVID-19. Figure 3 showed that IL-2R and IL-6 significantly decreased in cancer patients with better illness. These results strongly suggesting that the number of lymphocytes and cytokines could be used as independent prognostic factors to predict the outcome of cancer patients with COVID-19.

**Table 3:** Logistic regression analysis of survival of cancer patients tested for cytokines.

**Characteristics**	**All patients**	**P value**
**Age at diagnosis**	0.620 (0.179, 2.144)	0.401
**Gender**		0.016
Female	1 (Reference)	
Male	8.250 (1.044, 65.198)	
**Cancer type**		0.006
Non-hematologic cancer	1 (Reference)	
Hematologic cancer	5.380 (1.502, 19.269)	
**Comorbidities**		
Diabetes	1.343 (0.285, 6.328)	0.687
Hypertension	0.907 (0.234, 3.512)	0.756
Coronary heart disease	0.613 (0.150, 2.497)	0.331
COPD	1.254 (0.266, 5.907)	0.865
**Symptom**		
Dyspnea	11.556 (1.460, 91.474)	0.004
Cough	0.798 (0.169, 3.759)	0.865
Expectoration	4.855 (1.030, 22.898)	0.020
Malaise	1.365 (0.385, 4.840)	0.654
Headache	5.575 (0.455, 26.898)	0.060
Muscle ache	1.815 (0.230, 14.357)	0.650
Pharyngodynia	0.913 (0.116, 7.207)	0.877
Diarrhea	1.920 (0.542, 6.799)	0.153
Fever	2.179 (0.276, 17.202)	0.445
**Treatment**		
No	1 (Reference)	0.020
Yes	4.039 (1.043, 15.644)	
**Cytokines**		
TNF-α	4.510 (1.257, 21.259)	0.031
IL-2R	4.775 (1.012, 22.532)	0.031
IL-6	6.566 (1.497, 15.062)	<0.001
IL-8	4.811 (1.020, 22.687)	0.031
IL-10	8.201 (1.736, 38.748)	0.002

### Treatments and outcomes

Most cancer patients with COVID-19 (91.5%) received antivirus therapy, and 83.8% received intravenous antibiotic therapy; oxygen therapy was treated in 54.7% and mechanical ventilation in 28.1%; higher proportion of patients with critical disease received these therapies. Systemic glucocorticoids were given to 52 patients (44.4%). Patients with non-hematologic cancer did not die rapidly compared to patients with hematologic cancer types (odds ratio [OR] 5.380, 95% CI 1.502-19.269; p=0.006). Among cancer patients with COVID-19, those who underwent anti-cancer therapies in the past year had a markedly higher risk (33.3%) of death than did those not receiving anti-cancer therapies (15.0%) (OR 4.039, 95% CI 1.043-15.644; p=0.020).

## DISCUSSION

In this study, we exhibited clinical data of 117 cancer patients and paired non-cancer patients with nucleic acid-confirmed COVID-19. Similar to previous studies, fever and cough were the main symptoms and digestive symptoms were infrequent, which indicates a difference in viral tropism as compared to MERS-CoV, SARS-CoV, and seasonal influenza [8, 9].

A nationwide analysis indicated that COVID-19 patients with cancer had a poorer prognosis than those without cancer [10]. Our study demonstrated that cancer patients had more prominent laboratory abnormalities than non-cancer patients, accompanied with cancer patients seriously ill as compared to patients without a history of cancer [1, 10, 11]. 28 of the 117 cancer patients progressed to severe pneumonia as of March 30, 2020 and died during hospitalization. We showed that male, hematologic malignancies and dyspnea had been related to increased risk of death. Male sex is a well-known risk factor for COVID-19 patients [12]. Lymphocytopenia was common and associated with death, a finding that was in agreement with previous observations [13]. Lymphocytes play a critical role in the defense against infections. However, the effects induced by anti-cancer treatments contribute to the abnormal changes in amounts and functions of the immune cells in oncological population, which is more evident in some specific cancer type. Moreover, the heterogeneity of cancer cells and their interactions with cancer microenvironment can result in immunosuppression. So, the impact of preexistent immunological impairment in cancer patients with COVID-19 should be considered. Our data showed that a decrease of lymphocyte subsets was associated with higher mortality in cancer patients with COVID-19. Therefore, cancer patients who underwent anti-cancer therapies in the past year, had worse survival outcomes from COVID-19, alerting as a timely reminder to doctors that more attention should be paid to cancer patients, especially in male or those with specific cancer type [14].

Additionally, many cytokines also regulate the immune system and play a role in virus immunity. TNF α secretion promotes T lymphocyte proliferation and survival, whereas IL-2 levels reflect the T lymphocyte activation status toward a Th1 immune response [15]. IL-6 is involved in the differentiation of B lymphocytes into Ig-secreting cells and participates in lymphocyte and monocyte differentiation [16]. IL-8 is a chemotactic factor that attracts T lymphocytes, neutrophils, and basophils [17]. Previous studies have proved that elevated concentration of serum proinflammatory cytokines was related to pulmonary inflammation and respiratory failure in SARS patients [18]. MERS-CoV infection was also found to induce increased levels of proinflammatory cytokines [19]. In this study, cancer patients with higher concentration of TNF-α, IL-2R, IL-6, IL-8, and IL-10 had higher risk of death. In addition, we analyzed the alteration of major immune cell types (CD4^+^, CD8^+^ T cells, B cells and NK cells) in 117 cancer patients and showed that lymphocytes were diminished in cancer patients who died from COVID-19. This result suggests that COVID-19 might mainly act on immune cells, especially lymphocytes. Virus spreads through the respiratory and digestive mucosa and infects target cells, leads to a cytokine storm in severity illness cancer patients, generates the immune responses, and causes alteration in leukocytes and lymphocytes. Patients with severe illness developed acute respiratory distress syndrome and required mechanical ventilation. The decrease in the counts of lymphocytes suggests that the virus consumes the immune cells and blocks the immune function [20]. Lymphocyte deficiency might accelerate to exacerbations of patients [13]. In our study, IL-2R and IL-6 significantly decreased in the cancer patients who recovered from COVID-19. These results strongly suggesting that the number of cytokines and lymphocytes could be used as the prognostic factor to predict the outcome of cancer patients with COVID-19. Further studies that characterize the immune response in cancer patients with COVID-19 are required.

This study has a few notable limitations. Firstly, our study was undertaken in two designated hospitals in Wuhan, China. Large-scale multicenter clinical trials are needed to clarify our findings. Secondly, data collection was clinically driven and not systematic, some clinical records were missing, such as CT on admission, which helps to assess the role of cytokines and lymphocytes on organ injury. Thirdly, our cohort represented the more severe population of patients with COVID-19 since a series of asymptomatic patients and those who had mild symptoms were treated at home. However, bacterial coinfection was evident especially in the severe population. This might affect the response of the immune system. Finally, attention should also be paid to the long-term outcome of COVID-19 patients with cancer.

COVID-19 has spread rapidly since December 2019, and has been demonstrated to have a severe impact on human health. Moreover, compared to non-cancer patients, COVID-19 caused a higher proportion of multiple organ dysfunction syndrome in cancer patients which was linked to higher mortality (23.9%). Special attention should be given to these patients.

## MATERIALS AND METHODS

### Patients

In this retrospective, two-center study, we reviewed the medical records of all patients with laboratory-confirmed COVID-19 and cancer history who were hospitalized in Tongji hospital of Tongji Medical College, Huazhong University of Science and Technology and Renmin Hospital of Wuhan University. The admission date of these patients was from Jan 14, 2020 to March 12, 2020. Following the WHO interim guidance, COVID-19 was diagnosed by real-time quantitative reverse transcriptase–polymerase chain reaction (RT-PCR) assay or high-throughput sequencing of pharyngeal swab specimens. Nucleic acid confirmation of COVID-19 was conducted at the Chinese Center for Disease Prevention and Control until January 23, 2020, and subsequently in the two designated hospitals in Wuhan, China. Only nucleic acid-confirmed patients were incorporated into the analysis.

We extracted the clinical symptoms, comorbidities, and laboratory findings from electronic medical records. All the patients were followed up to April 20, 2020. If additional information or clarification was required, data were obtained directly from attending doctors or other health care providers. For each patient, the symptoms during their hospitalization were documented in the present study, including fever, cough, expectoration, fatigue, diarrhea, etc. Fever was defined as axillary temperature more than 37.5° C. All laboratory assessments were carried out depending on our institutional guidelines and clinical conditions of the patients. Laboratory testing included whole blood count, blood chemistry, coagulation test, lymphocyte subpopulation, and measures of procalcitonin, C-reactive protein (CRP), and serum cytokines. The primary composite end-point was death. The study was granted approval by the ethical committee of our institute with a waiver of written informed consent for emerging infectious diseases.

### Statistical analysis

Continuous variables were shown as median and compared by the Mann-Whitney U test; categorical variables were summarized as number (%) and compared by χ2 test or Fisher’s exact test between different groups. Scatter plots were drawn to describe lymphocyte subpopulation and plasma cytokine concentrations. The Kaplan-Meier method was utilized to measure the correlation of cytokines and lymphocytes with survival, and the Cox proportion hazard analysis was utilized to clarify potentially significant differences in outcome. A two-sided *p* value of less than 0.05 was considered significant for all applied statistical test. All the analyses were calculated with the use of R language, version 3.6.3 (http://www.r-project.org/).

### Ethics approval and consent to participate

The study is approved by the ethical committee of Tongji hospital, Tongji Medical College, Huazhong University of Science and Technology with a waiver of written informed consent for emerging infectious diseases.

### Availability of data and materials

The authors confirm that the data supporting the findings of this study are available within the article (and/or) its supplementary materials.

## Supplementary Material

Supplementary Figure 1

Supplementary Tables

## References

[1] Huang C, Wang Y, Li X, Ren L, Zhao J, Hu Y, Zhang L, Fan G, Xu J, Gu X, Cheng Z, Yu T, Xia J, et al. Clinical features of patients infected with 2019 novel coronavirus in Wuhan, China. Lancet. 2020; 395:497–506. 10.1016/S0140-6736(20)30183-5 31986264 PMC7159299

[2] Al-Abdallat MM, Payne DC, Alqasrawi S, Rha B, Tohme RA, Abedi GR, Al Nsour M, Iblan I, Jarour N, Farag NH, Haddadin A, Al-Sanouri T, Tamin A, et al, and Jordan MERS-CoV Investigation Team. Hospital-associated outbreak of Middle East respiratory syndrome coronavirus: a serologic, epidemiologic, and clinical description. Clin Infect Dis. 2014; 59:1225–33. 10.1093/cid/ciu359 24829216 PMC4834865

[3] Ksiazek TG, Erdman D, Goldsmith CS, Zaki SR, Peret T, Emery S, Tong S, Urbani C, Comer JA, Lim W, Rollin PE, Dowell SF, Ling AE, et al, and SARS Working Group. A novel coronavirus associated with severe acute respiratory syndrome. N Engl J Med. 2003; 348:1953–66. 10.1056/NEJMoa030781 12690092

[4] de Groot RJ, Baker SC, Baric RS, Brown CS, Drosten C, Enjuanes L, Fouchier RA, Galiano M, Gorbalenya AE, Memish ZA, Perlman S, Poon LL, Snijder EJ, et al. Middle East respiratory syndrome coronavirus (MERS-CoV): announcement of the coronavirus study group. J Virol. 2013; 87:7790–92. 10.1128/JVI.01244-13 23678167 PMC3700179

[5] Zhou P, Yang XL, Wang XG, Hu B, Zhang L, Zhang W, Si HR, Zhu Y, Li B, Huang CL, Chen HD, Chen J, Luo Y, et al. Addendum: A pneumonia outbreak associated with a new coronavirus of probable bat origin. Nature. 2020; 588:E6. 10.1038/s41586-020-2951-z 33199918 PMC9744119

[6] Kamboj M, Sepkowitz KA. Nosocomial infections in patients with cancer. Lancet Oncol. 2009; 10:589–97. 10.1016/S1470-2045(09)70069-5 19482247

[7] Scarcia M, Ludovico GM, Fortunato A, Fiorentino A. Patients with cancer in the COVID-19 era: the clinical trial issue. Tumori. 2020; 106:271–72. 10.1177/0300891620933672 32508257

[8] Chang D, Lin M, Wei L, Xie L, Zhu G, Dela Cruz CS, Sharma L. Epidemiologic and clinical characteristics of novel coronavirus infections involving 13 patients outside Wuhan, China. JAMA. 2020; 323:1092–93. 10.1001/jama.2020.1623 32031568 PMC7042871

[9] Assiri A, McGeer A, Perl TM, Price CS, Al Rabeeah AA, Cummings DA, Alabdullatif ZN, Assad M, Almulhim A, Makhdoom H, Madani H, Alhakeem R, Al-Tawfiq JA, et al, and KSA MERS-CoV Investigation Team. Hospital outbreak of Middle East respiratory syndrome coronavirus. N Engl J Med. 2013; 369:407–16. 10.1056/NEJMoa1306742 23782161 PMC4029105

[10] Liang W, Guan W, Chen R, Wang W, Li J, Xu K, Li C, Ai Q, Lu W, Liang H, Li S, He J. Cancer patients in SARS-CoV-2 infection: a nationwide analysis in China. Lancet Oncol. 2020; 21:335–37. 10.1016/S1470-2045(20)30096-6 32066541 PMC7159000

[11] Zhang Z, Li X, Zhang W, Shi ZL, Zheng Z, Wang T. Clinical features and treatment of 2019-nCov pneumonia patients in Wuhan: Report of a couple cases. Virol Sin. 2020; 35:330–36. 10.1007/s12250-020-00203-8 32034637 PMC7091133

[12] Xia Y, Jin R, Zhao J, Li W, Shen H. Risk of COVID-19 for patients with cancer. Lancet Oncol. 2020; 21:e180. 10.1016/S1470-2045(20)30150-9 32142622 PMC7130057

[13] Chen N, Zhou M, Dong X, Qu J, Gong F, Han Y, Qiu Y, Wang J, Liu Y, Wei Y, Xia J, Yu T, Zhang X, Zhang L. Epidemiological and clinical characteristics of 99 cases of 2019 novel coronavirus pneumonia in Wuhan, China: a descriptive study. Lancet. 2020; 395:507–13. 10.1016/S0140-6736(20)30211-7 32007143 PMC7135076

[14] Wang J, Zhang J, Tu Y, Zhou X, Huang H, Shao L, Chen L, Zhao Y, Ge M. Cancer patients in SARS-CoV-2 infection: a single-center experience from Wuhan. J Cancer. 2020; 11:6243–47. 10.7150/jca.47065 33033507 PMC7532501

[15] Chatzidakis I, Mamalaki C. T cells as sources and targets of TNF: implications for immunity and autoimmunity. Curr Dir Autoimmun. 2010; 11:105–18. 10.1159/000289200 20173390

[16] Larsen JV, Petersen CM. SorLA in interleukin-6 signaling and turnover. Mol Cell Biol. 2017; 37:e00641–16. 10.1128/MCB.00641-16 28265003 PMC5440653

[17] Schutyser E, Struyf S, Proost P, Opdenakker G, Laureys G, Verhasselt B, Peperstraete L, Van de Putte I, Saccani A, Allavena P, Mantovani A, Van Damme J. Identification of biologically active chemokine isoforms from ascitic fluid and elevated levels of CCL18/pulmonary and activation-regulated chemokine in ovarian carcinoma. J Biol Chem. 2002; 277:24584–93. 10.1074/jbc.M112275200 11978786

[18] Wong CK, Lam CW, Wu AK, Ip WK, Lee NL, Chan IH, Lit LC, Hui DS, Chan MH, Chung SS, Sung JJ. Plasma inflammatory cytokines and chemokines in severe acute respiratory syndrome. Clin Exp Immunol. 2004; 136:95–103. 10.1111/j.1365-2249.2004.02415.x 15030519 PMC1808997

[19] Mahallawi WH, Khabour OF, Zhang Q, Makhdoum HM, Suliman BA. MERS-CoV infection in humans is associated with a pro-inflammatory Th1 and Th17 cytokine profile. Cytokine. 2018; 104:8–13. 10.1016/j.cyto.2018.01.025 29414327 PMC7129230

[20] Liu WJ, Zhao M, Liu K, Xu K, Wong G, Tan W, Gao GF. T-cell immunity of SARS-CoV: implications for vaccine development against MERS-CoV. Antiviral Res. 2017; 137:82–92. 10.1016/j.antiviral.2016.11.006 27840203 PMC7113894

