# Supplementary material for: Lymphocyte may be a reference index of the outcome of cancer patients with COVID-19
Source: Aging (Albany NY). 2021 Mar 18;13(6):7733–44. doi: 10.18632/aging.202741 (PMC8034957; doi:10.18632/aging.202741)
Supplement: Supplementary Figure 1 [file aging-13-202741-s001.pdf]

SUPPLEMENTARY FIGURE

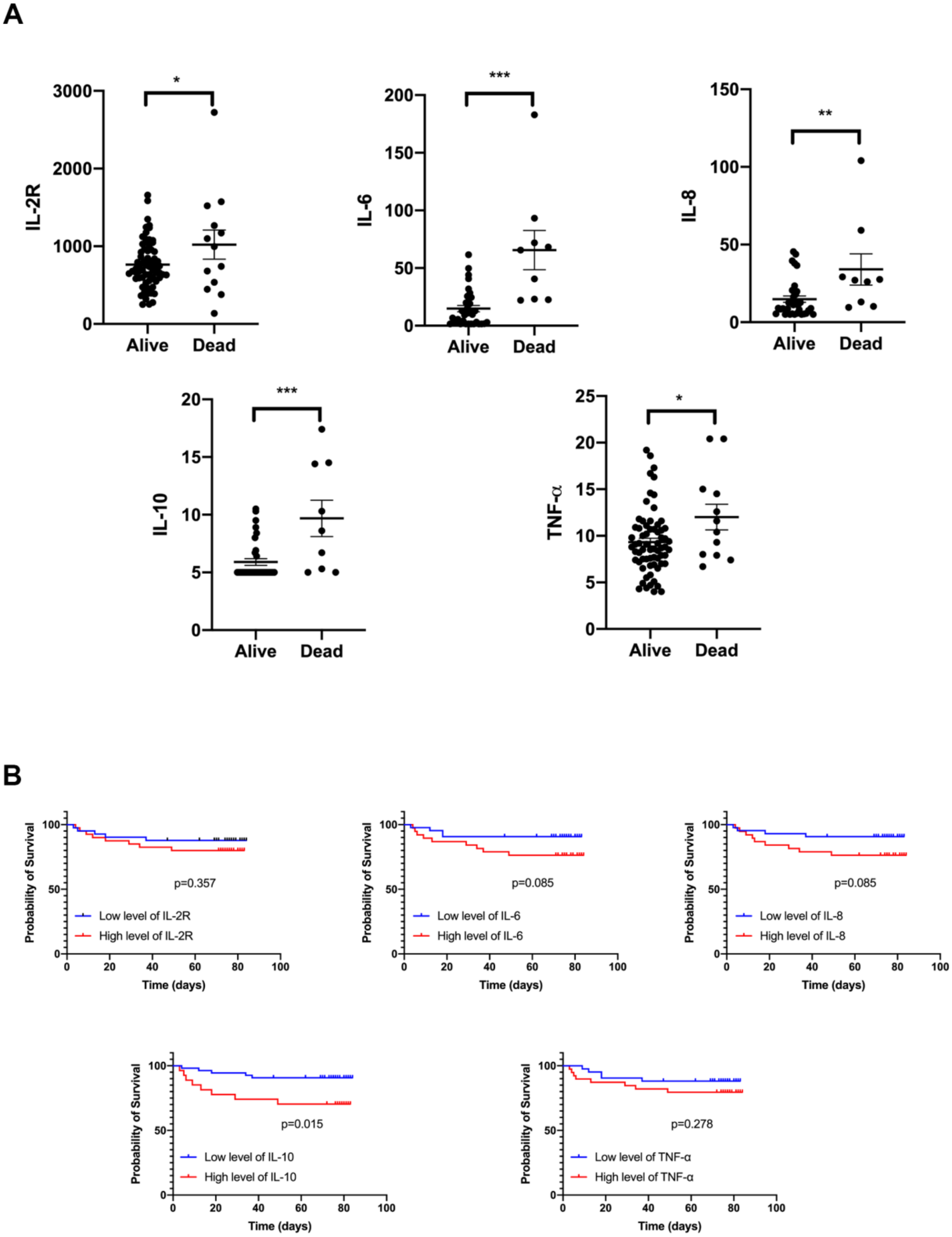

Supplementary Figure 1. Serum cytokines on admission of non-cancer patients with COVID-19. Levels of TNF-α, IL-2R, IL-6, IL-8, and IL-10 were increased in non-cancer patients who died of COVID-19 (**A**). The association between cytokines with survival time in non-cancer patients with COVID-19 (**B**).
