# Supplementary material for: Lymphocyte may be a reference index of the outcome of cancer patients with COVID-19
Source: Aging (Albany NY). 2021 Mar 18;13(6):7733–44. doi: 10.18632/aging.202741 (PMC8034957; doi:10.18632/aging.202741)
Supplement: Supplementary Tables [file aging-13-202741-s002.pdf]

SUPPLEMENTARY TABLES

Supplementary Table 1. Clinicopathological characteristics of non-cancer patients with COVID-19.

Characteristics	All patients (n=117)	Alive (n=98)	Dead (n=19)	P value
**Age (years)**				0.219
< 60	59 (50.4)	52 (53.1)	7 (36.8)	
≥ 60	58 (49.6)	46 (46.9)	12 (63.2)	
**Gender**				0.006
Female	59 (50.4)	55 (56.1)	4 (21.1)	
Male	58 (49.6)	43 (43.9)	15 (78.9)	
**Comorbidities**				
Diabetes	28 (23.9)	22 (22.4)	6 (31.6)	0.393
Hypertension	39 (33.3)	35 (35.7)	4 (21.1)	0.215
Coronary heart disease	16 (13.7)	11 (11.2)	4 (21.1)	0.241
**Symptom**				
Dyspnea	38 (32.5)	22 (22.4)	16 (84.2)	<0.001
Cough	97 (82.9)	81 (82.7)	16 (84.2)	0.869
Expectoration	51 (43.6)	43 (43.9)	7 (36.8)	0.571
Malaise	49 (41.9)	38 (38.8)	12 (63.2)	0.049
Headache	10 (8.5)	8 (8.2)	1 (5.3)	0.664
Muscle ache	30 (25.6)	25 (25.5)	4 (21.1)	0.680
Pharyngodynia	8 (6.8)	8 (8.2)	0	0.197
Diarrhea	31 (26.5)	31 (31.6)	0	0.004
Fever	104 (88.9)	85 (86.7)	18 (94.7)	0.325

Supplementary Table 2. Laboratory findings of non-cancer patients with COVID-19 on admission to hospital.

Characteristics	All patients (n=117)	Alive (n=98)	Dead (n=19)	P value
WBC, $\times 10^9$/L				
< 4	23 (27.7)	20 (28.6)	3 (23.1)	1
> 10	4 (4.8)	2 (2.9)	2 (15.4)	0.114
Neutrophil count, $\times 10^9$/L		3.33 (2.72, 4.26)	5.04 (3.35, 8.19)	0.002
Lymphocytopenia	60 (72.3)	48 (68.6)	12 (92.3)	0.100
Thrombocytopenia	2 (2.4)	1 (1.4)	1 (7.7)	0.290
Prothrombin time, s		14.1 (13.4, 14.9)	14.8 (13.5, 15.9)	1
APTT, s		41.4 (37.6, 45.1)	36.9 (33.8, 45.9)	1
D-dimer, mg/L		0.73 (0.45, 1.32)	1.25 (0.79, 7.29)	0.012
CRP		47.4 (10.4, 98.7)	87.6 (65.4, 158.2)	0.020
Procalcitonin		0.07 (0.04, 0.14)	0.20 (0.09, 0.30)	0.465
Total bilirubin		8.6 (6.3, 11.2)	11.5 (6.7, 13.5)	0.546
ALT > 40U/liter	19 (22.9)	14 (20.0)	5 (38.5)	0.162
AST > 40U/liter	29 (34.9)	20 (28.6)	9 (69.2)	0.009

Annotation: Lymphocytopenia was defined as a lymphocyte count of less than 1000 per cubic millimeter. Thrombocytopenia was defined as a platelet count of less than 100,000 per cubic millime- ter. APTT indicates activated partial thromboplastin time. AST indicates aspartate aminotransferase.
